# Establishment of Relational Model of Congenital Heart Disease Markers and GO Functional Analysis of the Association between Its Serum Markers and Susceptibility Genes

**DOI:** 10.1155/2016/9506829

**Published:** 2016-03-16

**Authors:** Min Liu, Luosha Zhao, Jiaying Yuan

**Affiliations:** ^1^Department of Cardiovascular Medicine, The First Affiliated Hospital of Zhengzhou University, No. 1 East Jianshe Road, Zhengzhou 450052, China; ^2^Department of Cardiovascular Medicine, Zhengzhou Central Hospital, Zhengzhou University, No. 195 Tongbai Road, Zhengzhou 450007, China; ^3^Department of Ultrasound Diagnosis, Directly under Hospital of Henan Military Region, No. 18 Jinshui Road, Zhengzhou 450000, China

## Abstract

*Purpose*. The purpose of present study was to construct the best screening model of congenital heart disease serum markers and to provide reference for further prevention and treatment of the disease.* Methods*. Documents from 2006 to 2014 were collected and meta-analysis was used for screening susceptibility genes and serum markers closely related to the diagnosis of congenital heart disease. Data of serum markers were extracted from 80 congenital heart disease patients and 80 healthy controls, respectively, and then logistic regression analysis and support vector machine were utilized to establish prediction models of serum markers and Gene Ontology (GO) functional annotation.* Results*. Results showed that NKX2.5, GATA4, and FOG2 were susceptibility genes of congenital heart disease. CRP, BNP, and cTnI were risk factors of congenital heart disease (*p* < 0.05); cTnI, hs-CRP, BNP, and Lp(a) were significantly close to congenital heart disease (*p* < 0.01). ROC curve indicated that the accuracy rate of Lp(a) and cTnI, Lp(a) and BNP, and BNP and cTnI joint prediction was 93.4%, 87.1%, and 97.2%, respectively. But the detection accuracy rate of the markers' relational model established by support vector machine was only 85%. GO analysis suggested that NKX2.5, GATA4, and FOG2 were functionally related to Lp(a) and BNP.* Conclusions*. The combined markers model of BNP and cTnI had the highest accuracy rate, providing a theoretical basis for the diagnosis of congenital heart disease.

## 1. Introduction

Congenital heart disease (CHD) indicates the presence of abnormality in heart and vascular structure and function at birth, the pathogenesis of which is complex. It is the interaction results of multiple factors like heredity and environment. The known risk factors include mental stimulation during pregnancy [1], harmful substances exposure [2], smoking and drinking [3], viral infections at early stage of pregnancy [4], diabetes mellitus [5], history of unhealthy pregnancy [6], and too high maternal age [7]. Its clinical consequences are extremely serious. It is the important cause of miscarriage, stillbirth, neonatal death, and children, adolescents, and adults with disabilities. The incidence of fetal CHD reaches as much as 6% to 10% [8] and continues to show a significant upward trend in China [9].

Currently, CHD is still cured by surgery. Many scholars believe that a number of indicators such as the level of serum C-reactive protein (CRP), brain natriuretic peptide (BNP), cardiac troponin I (cTnI), and Lipoprotein(a) (Lp(a)) can better reflect the functional status of the heart in patients with CHD and have good potential in clinical analysis. These proteins may serve as indicators in prognosis evaluation.

Since the United States has announced precision medicine plan, countries around the world have increased the support for precision medicine. With the enrichment and improvement of clinical big data and biological networks, it has become a general trend to complete interdisciplinary collaboration in disease prediction, diagnosis, and etiology analysis. In daily life, clinicians commonly use Logistic regression analysis to analyze the prognostic factors of the disease and estimate the probability of occurrence of variables [10]. Support vector machine (SVM) is a new machine learning method based on statistical theory. SVM is good at coping with linearly nonseparable sample data, which is achieved mainly through the slack variables (which are also called punishment variables) and kernel technology. It provides a unified framework in solving learning problems of finite samples [11].

Increasing studies show that the pathogenesis of congenital heart disease is related to certain transcription factors, while the relationship between the susceptibility genes and serological markers of congenital heart disease is not yet reported. With the rapid application of bioinformatics, Gene Ontology (GO) has become important tool and method in the field of bioinformatics. In terms of gene function annotation, GO plays a huge role. It can analyze the location of gene or protein in the cell, molecular functions, and biological processes involved; thus it simplifies the annotation of genes and their products as standardized vocabularies.

In this study, data of the susceptibility genes and clinical serology risk factors literatures of CHD were performed Meta-analysis to systematically evaluate them. By detecting levels of serum markers in patients with CHD, Logistic regression analysis, receiver operating characteristic (ROC) curve, and SVM approaches were used to evaluate the value of each serum marker in clinical diagnosis of CHD. The detection model of serum markers of this disease was then established. The functional relationship between susceptibility genes and serum markers was established by GO analysis. As a result, this study provides a theoretical basis for clinical practice and personalized treatment of cardiovascular disease.

## 2. Materials and Methods

### 2.1. Meta-Analysis

#### 2.1.1. Subjects

Clinical research documents on susceptibility genes and serological markers of CHD published in China and foreign countries from January 2006 to October 2014 were selected.

#### 2.1.2. Document Retrieval

Google Scholar was a major source of Chinese documents; PubMed, EMBASE, MEDLINE, and MD consult were main sources of English documents and the Chinese or English key words were “congenital heart disease”, “gene”, and “mutation” as well as “congenital heart disease”, “serum markers”, and “diagnosis”. The years of publication were from January 1, 2000, to October 31, 2014.

### 2.2. Statistical Analysis

RevMan5.1 was used for meta-analysis of the included literature. *p* ≥ 0.05 showed that the merge statistics of multiple studies had no statistical significance; *p* < 0.05 indicated that the combined statistics were statistically significant.

### 2.3. Establishing Relational Model of CHD Markers Group

#### 2.3.1. Research Data

In this study, 80 CHD patients (33 with atrial septal defect, 36 with ventricular septal defect, 3 with patent ductus arteriosus, and 8 with tetralogy of Fallot) received treatment in the Department of Cardiac Surgery at our hospital from December 2009 to September 2014 (54 males and 26 females, aged from 7 days to 59 years) and 80 healthy outpatients as determined by a physical examination given at the hospital (38 males and 42 females, aged 3.6 months to 51 years) were selected as the subjects. Patients in case group were confirmed by echocardiography and (or) surgery, and the following cases were excluded: (1) renal insufficiency, chronic liver disease, and acute and chronic infectious diseases; (2) systemic lupus erythematosus, rheumatoid, and other immune system diseases; and (3) infectious endocarditis, rheumatic heart disease, cardiac tumors, myocarditis, and other types of heart disease. Healthy control group denied a family history of CHD. They were confirmed to have no cardiac dysfunction and organic diseases by physical examination and echocardiography. Infection, trauma, autoimmune diseases, cancer, and so on were also excluded.

10 mL of venous blood was collected from all study subjects in the morning after 12 h overnight fasting and put into the EDTA anticoagulant tube. Samples were centrifuged within 2 h at 3 000 r/min for 10 min, and then the supernatants were collected.

#### 2.3.2. Sample Testing

Serum BNP level was detected using enzyme-linked immunosorbent assay (ELISA). Serum hs-CRP was examined using immune rate nephelometry. Immunofluorescence method was used to determine serum cTnI level. ELISA double-antibody sandwich assay was adopted to test serum Lp(a) level. Detection methods were carried out in strict accordance with the kit instructions. Each sample received parallel testing twice and the average value was regarded as final test results.

#### 2.3.3. Establishing Relational Model of CHD Markers Group Based on Logistic Regression Analysis

Serum markers BNP, hs-CRP, cTnI, and Lp(a) levels of CHD patients and healthy control group undergone Logistic regression analysis with the new variables of Logistic regression model as test variables and the pathological diagnosis results as state variables; the ROC curve was drawn. According to the value of the area under the curve (AUC) of ROC and diagnostic accuracy, its application value in early diagnosis of CHD was evaluated.

#### 2.3.4. Establishing Relational Model of CHD Markers Group Based on SVM

Data of the 80 CHD patients were treated with normalization processing. The establishment, training, and validation of SVM model were achieved through MATLAB programming.

#### 2.3.5. Statistical Analysis

The data obtained undergone significance of difference analysis using statistical software SPSS19.0 and the data were expressed by the following: mean ± standard deviation. *p* < 0.05 indicated that the difference was statistically significant.

### 2.4. Bioinformatics Functional Analysis of Serum Markers Lp(a) and BNP and Susceptibility Genes of CHD

#### 2.4.1. GO Retrieval

Congenital heart disease-related susceptibility genes NKX2.5, GATA4, and FOG2 and serological markers hs-CRP, Lp(a), BNP, and cTnI undergone GO functional annotation using AmiGO platform.

#### 2.4.2. RT-PCR

RNA kit from TAKARA (Takara Bio Inc., Shiga, Japan) was used to extract serum RNA, and Thermo Scientific RevertAid First Strand cDNA Synthesis Kit was used for reverse transcription experiments. With the synthesized cDNA template and GAPDH as template, we performed fluorescence quantitative PCR reactions. Fluorescent dye SYBR and quantitative real-time PCR instrument CFX96 were applied in this experiment. Primers are shown in Table 1 (primers were synthesized by Shanghai Sangon Biotech Co., Ltd., Shanghai, China). 20 *μ*L system of PCR reaction was as follows: 10 *μ*LSYBG Mix + 8 *μ*L H_2_O + 0.5 *μ*L upstream primer + 0.5 *μ*L downstream primer + 1 *μ*L cDNA; reaction conditions were as follows: denaturation at 95°C for 30 s, PCR reaction at 95°C for 5 s, and collecting fluorescence at 55°C for 30 s, with a total of 40 cycles, repeated three times.

## 3. Results

### 3.1. Meta-Analysis of Susceptibility Genes and Serum Markers

There were 176 documents about susceptibility and 216 documents about serum markers for initial survey after screening, there were 19 documents about susceptibility [12–31], and 20 documents about serum markers [32–51] were eventually included for meta-analysis.

Meta-analysis results of susceptibility genes and serum markers are shown in Tables 2 and 3. The heterogeneity test result of susceptibility genes NKX2.5 and FOG2 was *p* > 0.05, indicating the consistency of the literatures was well, so fixed effect model was used to pool the data. The heterogeneity test result of GATA4 was *p* < 0.05, suggesting that heterogeneity existed between the literatures, so the random effect model was adopted. The upper and lower limit of pooled SMD and 95% CI were greater than 1, indicating that the correlation between the mutation of three genes and congenital heart disease was statistical significance. The heterogeneity test result of three serum markers was *p* < 0.05, indicating that heterogeneity existed between literatures, so the random effect model was adopted. The upper and lower limit of pooled WMD and 95% CI were all greater than 0. Additionally, 95% CI transverse lines of three serum markers fell to the left side of the invalid vertical lines, suggesting that the incidence rate of the experimental group was bigger than that of the control group. Specific meta-analysis results are shown in Additional Files 1–6 (see Supplementary Material available online at http://dx.doi.org/10.1155/2016/9506829).

### 3.2. Test Results of Serum Markers

**Figure 1:**
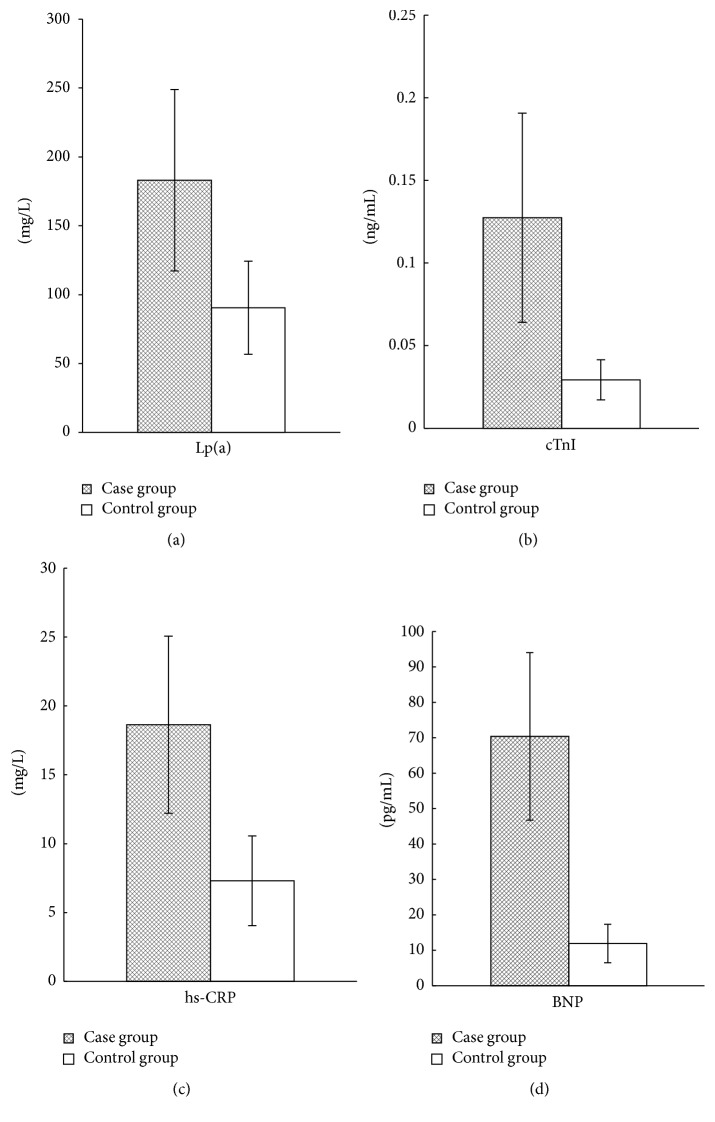
Detection results of four serum markers of CHD patients. *∗* indicates *p* < 0.05.

The test results of serum markers cTnI, hs-CRP, BNP, and Lp(a) of 80 patients with CHD and 80 healthy persons are shown in Figure 1. As can be seen from the figure, the levels of cTnI, hs-CRP, BNP, and Lp(a) in the case group were significantly higher than those in the controls (*p* < 0.05).

### 3.3. Logistic Regression Analysis Results

With cTnI, hs-CRP, BNP, and Lp(a) as independent variables and sick or not as the dependent variable, SPSS19.0 was used for dichotomy Logistic regression analysis. Univariate regression analysis results are presented in Table 4, which suggested that the relationship between Lp(a), BNP, and cTnI with CHD was statistically significant (*p* < 0.05). These three factors were then used for multivariate Logistic regression analysis. The results showed that the combination of these three factors was unfavorable for accurate diagnosis of CHD (*p* > 0.05, Table 5). Pairwise combinations of three factors were conducted for multivariate Logistic regression analysis and the results are presented in Table 6. It was indicated that the relationship between Lp(a), BNP, and cTnI with CHD had statistical significance (*p* < 0.05). The accuracy rates of combined predication of Lp(a) and cTnI, Lp(a) and BNP, and BNP and cTnI were 93.4%, 87.1%, and 97.2%, respectively.

### 3.4. Application Value Evaluation of Serum Markers on the Detection of CHD

**Figure 2:**
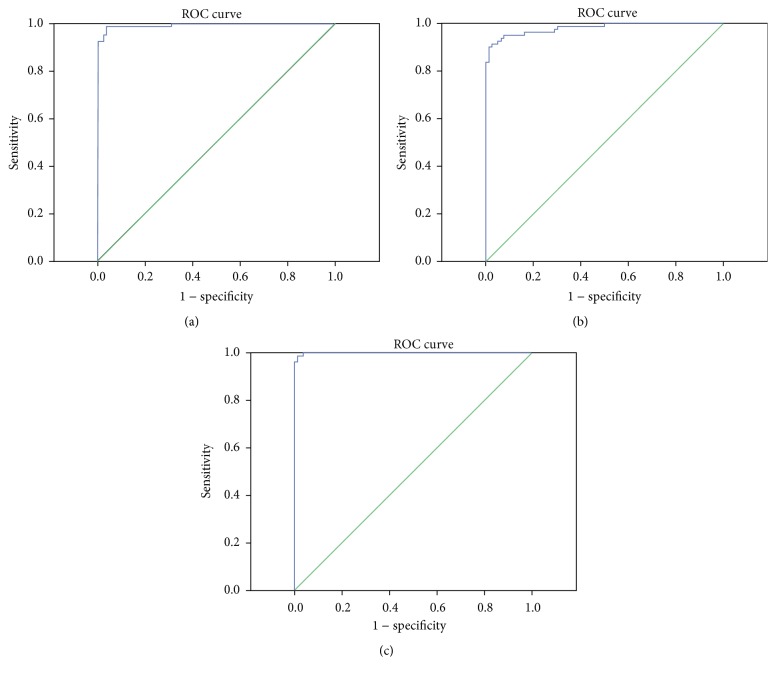
ROC curve of combined detection of CHD. (a) Lp(a) and cTnI; (b) Lp(a) and BNP; (c) BNP and cTnI.

SPSS19.0 software was adopted to evaluate the application value of Lp(a), BNP, and cTnI combined detection of CHD. ROC curves are shown in Figure 2. The AUC of Lp(a) and cTnI, Lp(a) and BNP, and BNP and cTnI joint detection were 0.994, 0.981, and 0.999, respectively, showing a high application value.

### 3.5. Establishing Relational Model of CHD Serum Markers Group Based on SVM

**Figure 3:**
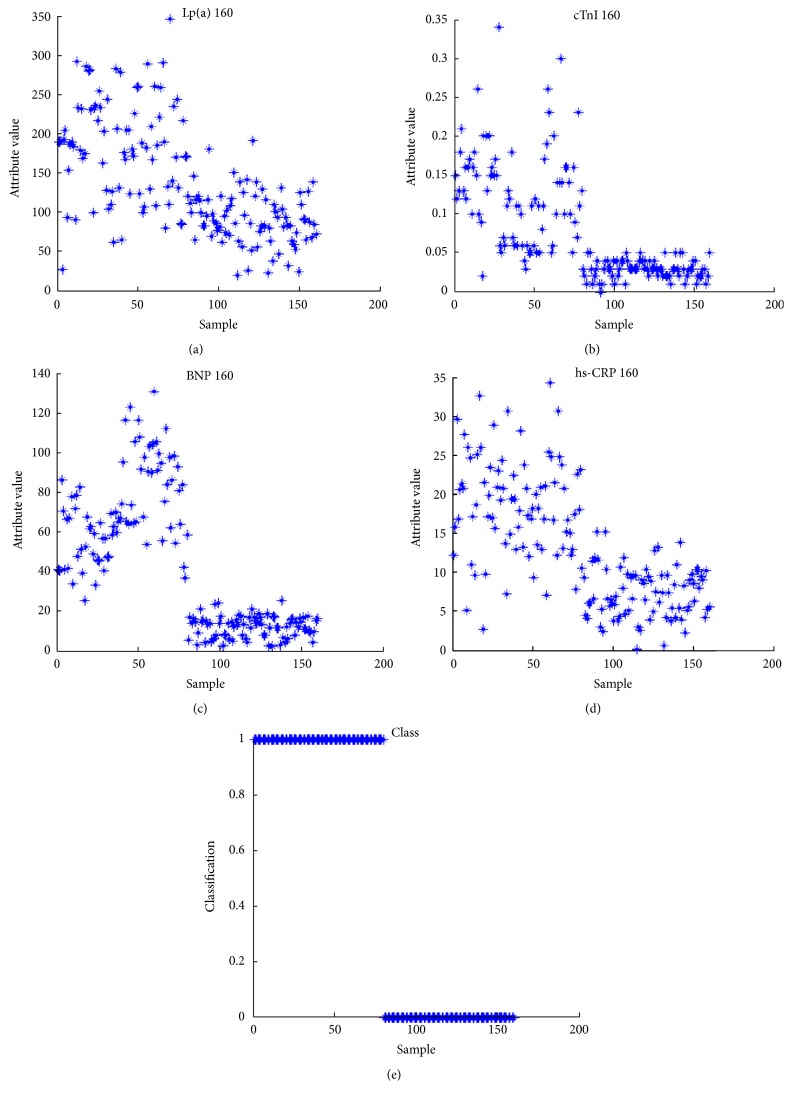
Attribution analyses of four serum markers of CHD.

Serum markers cTnI, hs-CRP, BNP, and Lp (a) levels of 80 CHD patients and 80 healthy controls undergone attributive analysis. It was indicated that attributive analysis had significant classification and the data were consistent with the basic calculation requirements of SVM (Figure 3).

**Figure 4:**
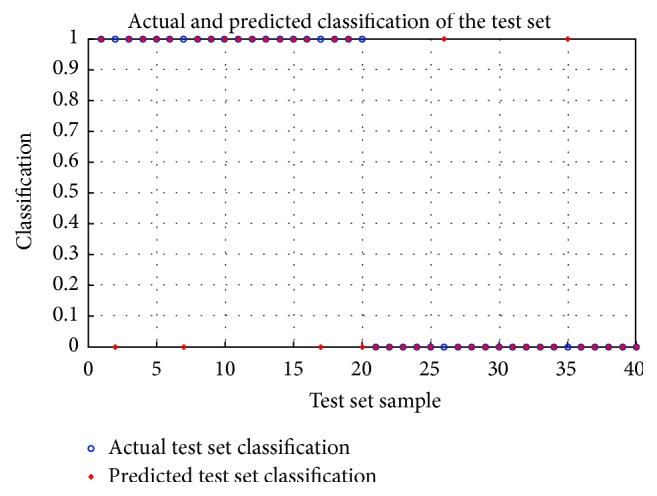
Relational model of CHD markers group based on SVM.

The relational model of CHD serum markers group based on SVM was established. Then, the test data of 20 CHD patients and 20 healthy controls were input into it. The test results are shown in Figure 4. The hollow circles represent the target output; “*∗*” is the actual simulation output of SVM. As can be seen from the figure, the diagnostic accuracy of the model was 34/40 = 85%.

### 3.6. GO Functional Annotation Results Comparison between Susceptibility Genes and Serum Markers of CHD

After comparing the GO functional annotation results of susceptibility genes NKX2.5, GATA4, and FOG2 and serological indicators hs-CRP, Lp(a), BNP, and cTnI, it was found that NKX2.5, GATA4, and FOG2 had same GO functional annotation with Lp(a) and BNP. The functional relations between three susceptibility genes and BNP were mainly in gene expression and metabolic process. The internal connections between Lp(a) and NKX2.5, GATA4, and FOG2 were mainly in function, especially in the aspects of Lipoprotein transmembrane transport and blood circulation. The same GO functional annotations of them are shown in Tables 7 [Table tab8]–9.

### 3.7. Relative Expression Contents of Susceptibility Genes in mRNA Level

**Figure 5:**
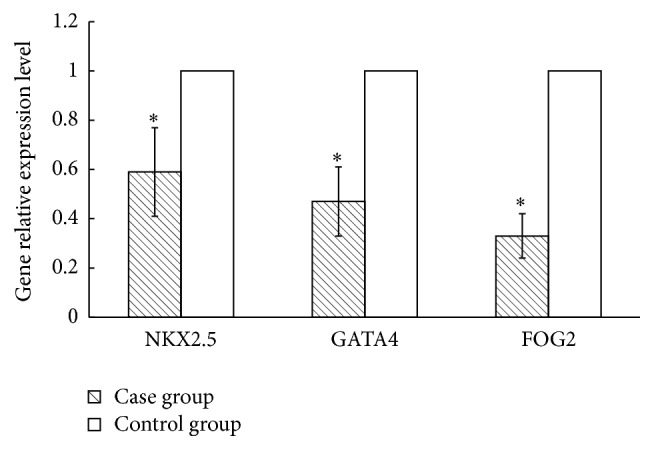
The relative expression levels of CHD susceptibility genes. *∗* indicates *p* < 0.05.

Real-time fluorogenic quantitative PCR was used to detect the expression levels of susceptibility genes NKX2.5, GATA4, and FOG2 in mRNA. 2^−ΔΔCt^ was used to calculate the relative expression levels, and the results were 0.59 ± 0.18, 0.47 ± 0.14, and 0.33 ± 0.09, respectively. If the content of the control group was 1, the relative expression levels of NKX2.5, GATA4, and FOG2 in the case group were 0.59 ± 0.18, 0.47 ± 0.14, and 0.33 ± 0.99, respectively (Figure 5). The expression levels of susceptibility genes NKX2.5, GATA4, and FOG2 in the case group were obviously lower than those in the controls. The results of serum indexes detection showed that Lp(a) and BNP levels in the case group were significantly higher than those in the controls (Figure 1). Thus it can be inferred that the unusual increase of serum Lp(a) and BNP levels may be related to the abnormal expression of NKX2.5, GATA4, and FOG2 genes.

## 4. Discussion

CHD is the most common congenital malformation at present and also the leading cause of infant death. Many factors interact with each other temporally and spatially in the development of heart. The combined actions of hereditary and environmental factors in embryonic phase will lead to the dysplasia of heart. Due to the complex genetic mechanism of CHD, the reason resulting in the malformation of heart is still unclear. The type of CHD is diverse, which has become a big problem in the treatment and prevention of CHD.

In this study, meta-analysis found that the mutation of NKX2.5, GATA4, and FOG2 genes played an important role in the development of CHD. The mutation of NKX2.5 gene occurred mainly in homeodomain structural domain. McElhinney et al. [52] reported that the mutation of exon 1 of NKX2.5 gene existed in various CHD. The pathological and physiological effects of GATA4 gene related to heart development have been extensively researched. Garg et al. [53] have verified that GATA4 gene mutation is one of the causes of CHD for the first time by the molecular genetics research on two independent and simple CHD families. FOG2 gene is a transcription factor with early expression in the process of heart development. Its interaction with GATA4 runs through the entire process of heart development. FOG2 plays an essential role in the development process of heart [3]. Both Tan and De Luca found a mutation in FOG2 gene exon from patients with double-outlet right ventricle combined ventricular septal defect [30, 31]. This paper found that serum markers cTnI, hs-CRP, and BNP were related to CHD and they can predict the occurrence of the disease. Guo [32] believed that the changes in serum levels of cTnI were of great value in understanding the state and prognosis of CHD. However, researches on the relationship between Lp(a) and CHD were much rare, and Lp(a) did not meet the condition of meta-analysis, so we could not perform analysis of this factor.

By examining the levels of cTnI, hs-CRP, BNP, and Lp(a) of 80 CHD patients and 80 healthy control subjects, this study showed that the levels of cTnI, hs-CRP, BNP, and Lp(a) in the case group were significantly higher than those in the controls, and the difference was statistically significant. Geiger et al. [54] found that, compared to the non-CHD subjects, BNP level of CHD children was obviously increased.. Similarly, Akhabue et al. [55] also believe that the difference of BNP concentration between CHD children patients and non-CHD children was significant. A number of studies show that the relationship between LP(a) and atherosclerotic disease was close, and the increased LP(a) is an independent risk factor of cardiovascular events [56–59]. Guo [32] has shown that serum cTnI level in patients with CHD was significantly higher than that in normal people. Logistic regression analysis showed that there existed significant correlations between cTnI, BNP, Lp(a), and CHD. When performing combined diagnosis, cTnI, BNP, and Lp(a) pairwise binding were associated with CHD. According to the joint detection ROC curve, it was found that the pairwise combination AUC of cTnI, BNP, and Lp(a) were greater than 0.9, and the accuracy rates were higher than 87%. The bigger the data is, the better the effect is when using Logistic regression model. SVM in contrast has a higher accuracy rate as to small sample size.

Recent studies showed that GATA4 and GATA6 can collaborate and regulate the expression of brain natriuretic peptide (BNP). The deletion of any factor of GATA will lead to the downregulation of BNP level [60]. Other studies indicated that NKX2.5 and FOG2 could cooperate with GATA4, all of which play an important role in the normal process of heart development [61, 62]. As an independent protein molecule having a specific antigenicity, the metabolic pathways of Lp(a) is completely different from other apolipoproteins. It can interfere with lipid metabolism and the fibrinolytic system and then play an important role in cardiovascular diseases like thrombosis and atherosclerosis [63, 64]. Studies have shown that Lp(a) is an independent risk factor for myocardial infarction, coronary heart disease, and other cardiovascular diseases [65–68], but few researches are conducted on the relationship between Lp(a) and CHD. At present, it is not reported which transcription factor Lp(a) is regulated by. By bioinformatics analysis, this study showed that there were the same GO functional annotations between susceptibility gene NKX2.5, GATA4, and FOG2 and Lp(a) and BNP. The links between susceptibility genes and BNP existed mainly in gene expression and metabolism. Lp(a), especially in Lipoprotein membrane transport and blood circulation, was intrinsically linked to NKX2.5, GATA4, and FOG2. This paper conducted a study on the mRNA relative expression levels of susceptibility genes, Lp(a) and BNP. It was indicated that the levels of NKX2.5, GATA4, and FOG2 of the case group were significantly lower than those of the controls. The contents of Lp(a) and BNP of the case group were significantly higher than those of the controls, suggesting that the abnormal expression of susceptibility genes may lead to the increase of BNP level. However, the mechanism which causes the abnormal expression of Lp(a) is still not clear, so further study is required. This also gives us a direction on the in-depth study of CHD.

## 5. Conclusions

In conclusion, as risk factors associated with CHD, cTnI, CRP, BNP and Lp(a) also have functional relation with susceptibility genes; therefore, they may provide a basis for the clinical detection of CHD, but its specific application still requires a lot of clinical cases data to train and optimize, thus making it more accurate. Clinical auxiliary testing model is only as an auxiliary tool at the early stage and cannot completely replace an experienced clinician's diagnosis. The clinical diagnosis of CHD still needs to integrate all aspects of judgments.

## Supplementary Material

Additional files 1, 2 and 3 are Meta-analysis of NKX2.5, GATA4 and FOG2. Files 4, 5 and 6 are Meta-analysis results of serum cardiac troponie, high-sensitivity C-reactive protein and BNP. All of these aims to screen genes and proteins closely related to congenital heart disease.

## Figures and Tables

**Table 1:** Name, sequence, and product length of each primer.

Primer name	Primer sequence	Product length
NKX2.5-F	AGAAGACAGAGGCGGACAAC	175 bp
NKX2.5-R	CGTGGACGTGAGTTTCAGCA	

GATA4-F	GTGTCCCAGACGTTCTCAGT	226 bp
GATA4-R	TCCGTGCAGGAATTTGAGGA	

FOG2-F	TTAATCAACGGAAGCAAATG	466 bp
FOG2-R	CCACTCAAATACAGGGTTAGG	

GAPDH-F	AGAAGGCTGGGGCTCATTTG	258 bp
GAPDH-R	AGGGGCCATCCACAGTCTTC	

**Table 2:** Meta-analysis results of susceptibility genes.

Susceptibility gene	Number of documents	Heterogeneity test		OR	95% CI
		*I* ^2^	*p*		
NKX2.5	7	36%	0.16	2.02	1.42–2.86
GATA4	11	58%	0.01	2.08	1.50–2.88
FOG2	4	0%	0.85	19.43	4.52–83.63

**Table 3:** Meta-analysis of serum markers.

Serum markers	Number of documents	Heterogeneity test		WMD	95% CI
		*I* ^2^	*p*		
cTnI	4	99.2%	0.000	0.33	0.12~0.55
hs-CRP	8	87.5%	0.000	1.84	1.36~2.32
BNP	19	99.9%	0.000	321.33	279.03–364.29

**Table 4:** Univariate regression analysis of serum markers associated with CHD.

Serum markers	*B*	SE	Wals	Sig.	Exp(*B*)
Lp(a)	0.036	0.006	40.422	0.000	1.036
BNP	0.478	0.077	38.954	0.000	1.612
hs-CRP	83.858	400.679	0.044	0.834	2.625*E*36
cTnI	172.096	37.484	21.079	0.000	5.502*E*74

**Table 5:** Three-factor multivariate regression analysis of serum markers associated with CHD.

Serum markers	*B*	SE	Wals	Sig.	Exp(*B*)
Lp(a)	0.046	52.695	0.018	0.869	1.047
BNP	156.787	72890.460	0.008	0.924	1.235
cTnI	0.665	152.952	0.000	0.997	1.944

**Table 6:** Two-factor multivariate regression analysis of serum markers associated with CHD.

	Serum markers	*B*	SE	Wals	Sig.	Exp(*B*)
1	Lp(a)	0.045	0.011	15.672	0.000	1.046
	BNP	0.508	0.114	19.774	0.000	1.662

2	Lp(a)	0.043	0.012	11.876	0.001	1.044
	cTnI	211.657	61.321	11.914	0.001	8.348*E*91

3	BNP	0.682	0.247	7.619	0.006	1.978
	cTnI	263.631	111.283	5.612	0.018	3.115*E*114

**Table 7:** The same GO semantic annotation of NKX2.5 gene and serum markers Lp(a) and BNP.

Gene	GO number	Annotation	Ontology	Evidence	Reference	Serum marker
NKX2.5	0010467	Gene expression	Biological process	TAS	Reactome: REACT_71	Brain natriuretic peptide (BNP)
	0006367	Transcription initiation from RNA polymerase II promoter	Biological process	TAS	Reactome: REACT_118713 Reactome: REACT_12627	
	0007166	Cell surface receptor signaling pathway	Biological process	NAS	PMID: 12727915	
	0008015	Blood circulation	Biological process	TAS	PMID: 8047165	Lipoprotein(a) (Lp(a))
	0055085	Transmembrane transport	Biological process	TAS	Reactome: REACT_15480	
	0007399	Nervous system development	Biological process	IEA: with Ensemb ENSMUSP00000036044	GO REF: 0000019	

**Table 8 tab8:** The same GO semantic annotation of FOG2 gene and serum markers Lp(a) and BNP.

Gene	GO number	Annotation	Ontology	Evidence	Reference	Serum marker
FOG2	0001701	In utero embryonic development	Biological process	IEA: with Ensemb ENSMUSP00000036044	GO REF: 0000019	Brain natriuretic peptide (BNP)
	0007596	Blood coagulation	Biological process	TAS	Reactome: REACT_604	
	0010467	Gene expression	Biological process	TAS	Reactome: REACT_71	Lipoprotein(a) (Lp(a))
	0044702	Single organism reproductive process	Biological process	IEA: with Ensemb ENSMUSP00000027449l	GO REF: 0000019	

**Table 9:** The same GO semantic annotation of GATA4 gene and serum markers Lp(a) and BNP.

Gene	GO number	Annotation	Ontology	Evidence	Reference	Serum marker
GATA4	0010467	Gene expression	Biological process	TAS	Reactome: REACT_71	Brain natriuretic peptide (BNP)
	0007166	Cell surface receptor signaling pathway	Biological process	NAS	PMID: 12727915	
	0014898	Cardiac muscle hypertrophy in response to stress	Biological process	IEA: with Ensemb ENSMUSP000000099520	GO REF: 0000019	
	0044702	Single organism reproductive process	Biological process	IEA: with Ensemb ENSMUSP00000027449l	GO REF: 0000019	
	0008015	Blood circulation	Biological process	TAS	PMID: 8047165	Lipoprotein(a) (Lp(a))
	0001701	In utero embryonic development	Biological process	IEA: with Ensemb ENSMUSP00000036044	GO REF: 0000019	
	0007283	Spermatogenesis	Biological process	IEA: with Ensemb ENSMUSP00000036044	GO REF: 0000019
	0009743	Response to carbohydrate	Biological process	IEA: with Ensemb ENSRNOP00000039779	GO REF: 0000019
