# Supplementary material for: Establishment of Relational Model of Congenital Heart Disease Markers and GO Functional Analysis of the Association between Its Serum Markers and Susceptibility Genes
Source: Comput Math Methods Med. 2016 Mar 16;2016:9506829. doi: 10.1155/2016/9506829 (PMC4812235; doi:10.1155/2016/9506829)

Additional file 1 Forest map of Meta analysis of the influence of NKX2.5 gene on CHD


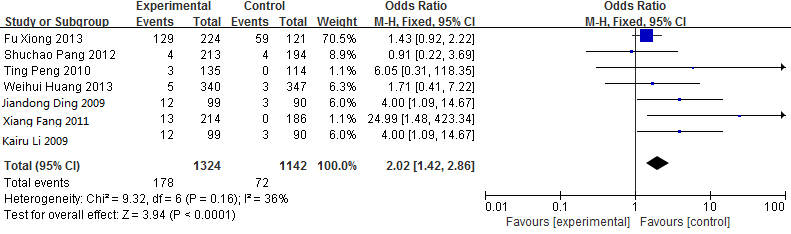


Additional file 2 Forest map of Meta analysis of the influence of GATA4 gene on CHD


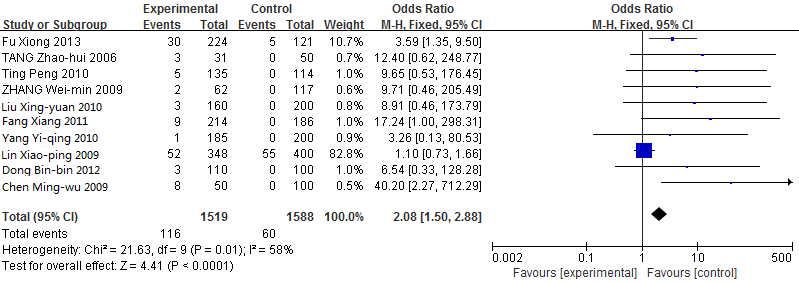


Additional file 3 Forest map of Meta analysis of the influence of FOG2 gene on CHD
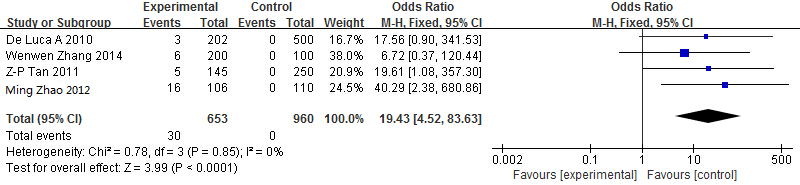


Additional file 4 Meta analysis of cardiac troponie


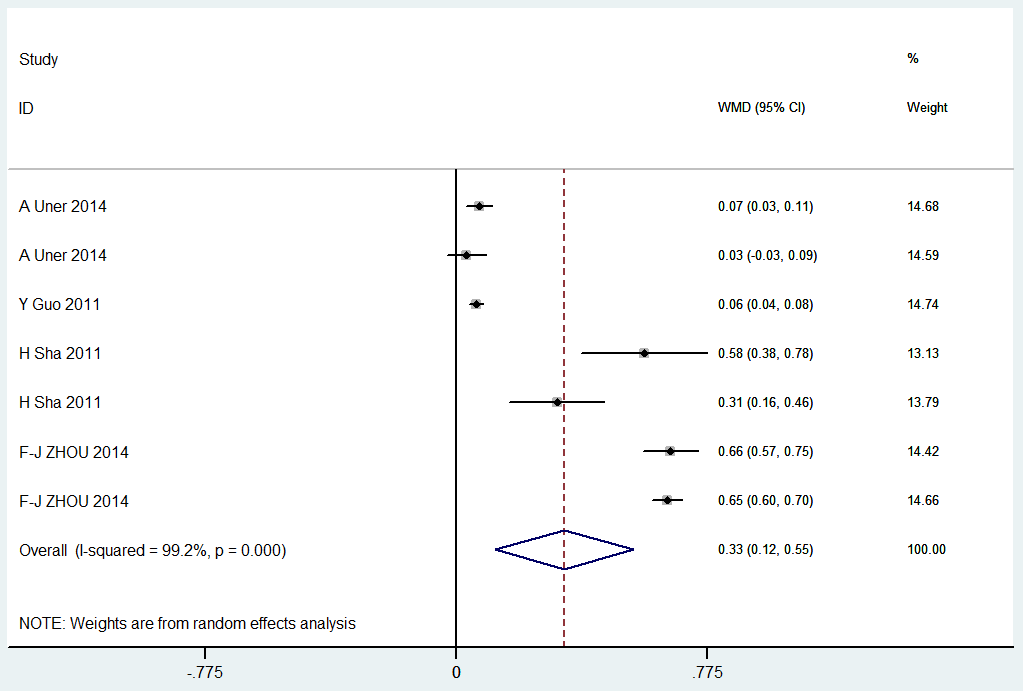


Additional file 5 Meta-analysis results of high-sensitivity C-reactive protein


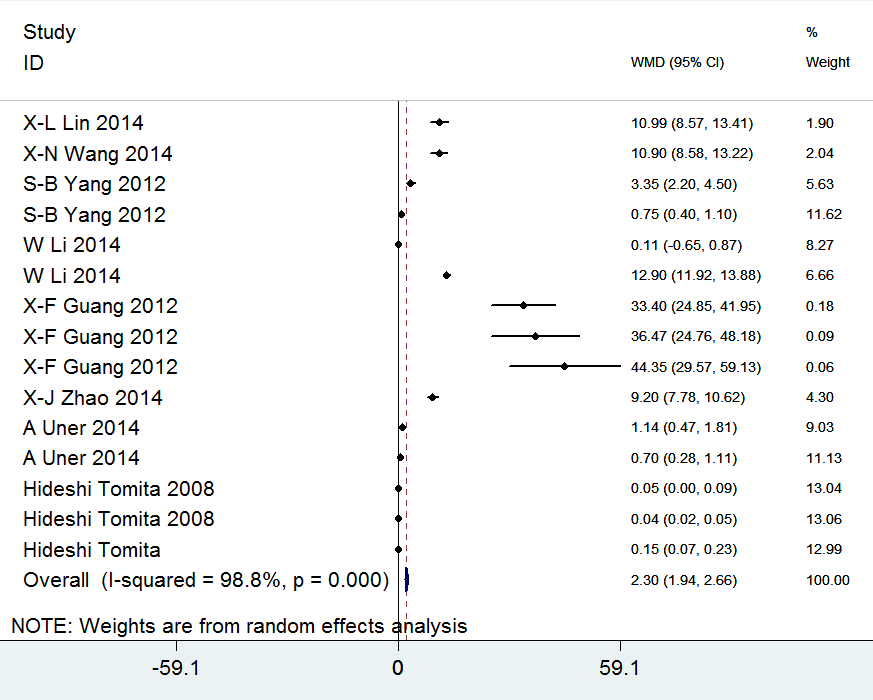


Additional file 6 Meta analysis results of BNP


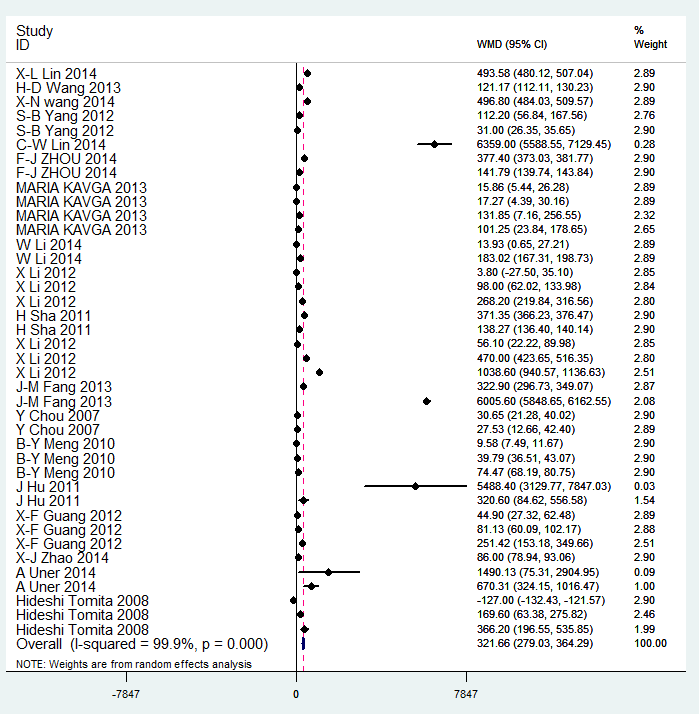

Supplement: Supplementary file 1 — Additional files 1, 2 and 3 are Meta-analysis of NKX2.5, GATA4 and FOG2. Files 4, 5 and 6 are Meta-analysis results of serum cardiac troponie, high-sensitivity C-reactive protein and BNP. All of these aims to screen genes and proteins closely related to congenital heart disease. [file 9506829.f1.doc]
